# Insights into an evolutionary strategy leading to antibiotic resistance

**DOI:** 10.1038/srep40357

**Published:** 2017-01-11

**Authors:** Chun-Feng D. Hou, Jian-wei Liu, Charles Collyer, Nataša Mitić, Marcelo Monteiro Pedroso, Gerhard Schenk, David L. Ollis

**Affiliations:** 1 Research School of Chemistry, The Australian National University, Canberra, ACT 0200, Australia; 2 CSIRO Entomology, Black Mountain, ACT 2601, Australia; 3 School of Molecular Bioscience, The University of Sydney, NSW 2006, Australia; 4 Department of Chemistry, Maynooth University, Maynooth, Co. Kildare, Ireland; 5 School of Chemistry and Molecular Biosciences, The University of Queensland, Brisbane, QLD 4072, Australia

## Abstract

Metallo-β-lactamases (MBLs) with activity towards a broad-spectrum of β-lactam antibiotics have become a major threat to public health, not least due to their ability to rapidly adapt their substrate preference. In this study, the capability of the MBL AIM-1 to evade antibiotic pressure by introducing specific mutations was probed by two alternative methods, *i.e*. site-saturation mutagenesis (SSM) of active site residues and *in vitro* evolution. Both approaches demonstrated that a single mutation in AIM-1 can greatly enhance a pathogen’s resistance towards broad spectrum antibiotics without significantly compromising the catalytic efficiency of the enzyme. Importantly, the evolution experiments demonstrated that relevant amino acids are not necessarily in close proximity to the catalytic centre of the enzyme. This observation is a powerful demonstration that MBLs have a diverse array of possibilities to adapt to new selection pressures, avenues that cannot easily be predicted from a crystal structure alone.

It was not until the early 1940 s that a highly effective agent to combat bacterial infections was introduced, penicillin. However, it soon emerged that bacteria are able to resist antibiotics in a number of ways^1^,^2^,^3^. One of their most efficient strategies involves the synthesis of enzymes that degrade the antibiotics. There are four different types of enzymes that inactivate most of the derivatives of penicillin commonly used in medical applications (Fig. 1). These β-lactamases are divided into four groups. Groups A, C and D bear structural and mechanistic similarity to penicillin-binding proteins and can be inhibited by drugs such as clavulanic acid^4^. Group B enzymes are distinct from the other β-lactamases in that they require Zinc(II) ions for catalytic activity^5^,^6^,^7^. Such metallo-β-lactamases (MBLs) were first observed in the 1960 s, but have become common in recent years^5^,^6^,^7^,^8^,^9^. MBLs have been further divided into several subclasses based on sequence variations and their requirement for zinc^5^,^6^,^7^,^10^,^11^,^12^. Members of the B1 and B3 subgroups operate optimally as bimetallic enzymes, whereas those from the B2 subgroup only need one Zn(II) for catalytic activity; the presence of a second metal ion leads to an inhibited state^13^,^14^. More recently, a fourth subgroup was proposed, based on the observation that some MBLs may be in a catalytically inactive mononuclear resting state and only upon addition of a substrate an active bimetallic centre is formed^15^,^16^.

AIM-1 is a member of the B3 subgroup and was initially identified in a sample from a patient suffering from an antibiotic-resistant infection from *Pseudomonas aeruginosa*; the enzyme has since been shown to have a broad spectrum of activity toward most clinically relevant β-lactams^17^,^18^. In terms of sequence homology, AIM-1 is most similar to the B3-type MBL SMB-1 from the carbapenem-resistant *Serratia marcescens*, with the two enzymes sharing 55% identity^19^,^20^. More recently, enzymes homologous to both AIM-1 and SMB-1 have also been identified in environmental microorganisms *Novosphingobium pentaromativorans (i.e*. MIM-1) and *Simiduia agarivorans (i.e*. MIM-2)^21^. While both MIM-1 and MIM-2 are efficient MBLs^21^,^22^ they are also very potent lactonases^23^, indicating that a similar active site structure may accommodate rather diverse substrates. The gene encoding AIM-1 is located in the chromosome and, similar to SMB-1, is flanked by two copies of ISCR elements that are implicated in the mobilisation of several MBL-encoding genes^17^,^19^,^24^. These mobile elements may accelerate the spread of resistance genes among clinically opportunistic pathogens, enhancing the threat of AIM-1 to health care.

The present study was motivated by a desire to obtain functional insight that might be useful in drug design. Specifically, we wanted to identify (i) ways in which MBLs might evolve to promote antibiotic resistance and (ii) residues that are crucial in facilitating this adaptation. Since AIM-1 has a broad specificity towards a wide range of substrates we employed *in vitro* evolution techniques to identify residues that may be essential for the function of this enzyme and to demonstrate how AIM-1 can adapt to β-lactam substrates. While this is the first study to probe the adaptability of a MBL from the B3 subgroup similar techniques have been employed to probe the mechanism and evolution of the well-characterised B1-type MBLs IMP^24^,^25^,^26^,^27^ and BcII^28^,^29^,^30^,^31^, as well as the carbapenem-specific B2-type MBL CphA^32^,^33^. Specifically, we used site-saturation mutagenesis (SSM) to generate mutant forms of AIM-1 and screened the mutants for enhanced activity and altered substrate specificity. A total of 85 mutants were generated with two highly efficient mutants displaying enhanced activity towards representatives of each of the three major substrate groups (*i.e*. penams, cephalosporins and carbapenems).

## Results and Discussion

In this study two approaches to alter the substrate specificity of the antibiotic-degrading metallohydrolase AIM-1 were compared, (i) structure-guided protein engineering using SSM, and (ii) *in vitro* evolution driven by increasing the concentration of a particular substrate (*i.e*. antibiotic).

### Site-saturation mutagenesis

The residues to be mutated were selected based on their proximity to the catalytic, Zn(II)-containing centre of the enzyme (Fig. 2). Mutation-introducing oligonucleotides were of the form NNK at the codon of the amino acid to be altered so that, in principle, all possible amino acids may be represented in the library of mutant proteins. We selected 80 mutants at random covering between three (*i.e*. position T87) to 15 (*i.e*. position Q157) mutations (Table 1).

The number of mutations that led to positive, negative or negligible (neutral) changes in the activity towards the three types of substrates tested is shown in Fig. 3. The majority of the mutations had a minimal effect on the activity towards the substrate ampicillin. Only replacements at position 221 were predominantly detrimental. The effects of the mutations were more significant when cefoxitin and imipenem were used as substrates, with both detrimental and advantageous mutations being observed. While most mutations have a less detrimental effect towards the reactions with cefoxitin and imipenem, the notable exceptions are mutations in position 157 – while only 3 of the 15 mutations have a negative effect on the reaction with ampicillin, the majority of mutations in this site are detrimental towards the reactions with cefoxitin and imipenem. Also, most mutations in position 221 are detrimental for the enzyme independent of the substrate used in the assays.

S221 is conserved among the B3-type MBLs and according to crystallographic and mutagenesis studies of AIM-1, L1, FEZ-1 and SMB-1 its hydroxyl group may form a hydrogen bond with a bound substrate molecule^19^,^20^,^34^,^35^,^36^. Interestingly, the corresponding residue in B1- and B2-type MBLs is C221, a ligand of one of the Zn(II) ions in the active site^5^,^6^,^7^. Two mutations in position 221 were selected for a more detailed catalytic characterization (Table 2). Removing the possibility of a hydrogen bonding interaction (*i.e*. S221A mutation) had a slightly negative effect on the reaction with ampicillin and a largely positive impact on the reaction with cefotoxin, but was strongly deleterious on the reaction with imipenem (although substrate binding was improved, judged by a reduction in the corresponding *K*_*m*_ values). In contrast, introducing a bulkier negative charge (*i.e*. S221E mutation) destroyed the enzyme’s capability to hydrolyse any of the three substrates. Thus, position 221 plays an important role in regulating enzymatic efficiency and substrate selectivity.

While it is no trivial task to assign a particular side chain to specific functions of an enzyme-catalysed reaction the example above demonstrated that S221 plays an important role in substrate recognition. Another relevant example is residue F119. Similar to position 157, this site is fairly resistant to mutations as long as the substrate ampicillin was used to assess catalytic activities (Fig. 3). In contrast, the same site is more susceptible to mutations when cefoxitin or imipenem were used, with some mutations leading to improved and others to detrimental changes in catalytic performance. One particular mutation, F119M, leads to a significant improvement for the hydrolysis of cefoxitin, mainly by lowering the *K*_*m*_ value by one order of magnitude (Table 2). The same mutation has virtually no effect on the *k*_*cat*_ and *K*_*m*_ of the reaction with imipenem while ampicillin is hydrolysed considerably faster at the cost of less favourable substrate binding. The net result is that the specificity of AIM-1 is altered in a way that the mutant form of the enzyme displays a similar preference for all three types of substrates.

### Directed evolution of AIM-1

The above study demonstrated that single mutations on residues in the vicinity of the catalytic centre of AIM-1 have the potential to affect the substrate specificity of the enzyme. In order to probe the ability of AIM-1 to adapt to different substrates further error-prone PCR (ePCR) was used to generate mutant libraries over four rounds. The average mutation rate of these libraries is 0.67% and 0.3% per amino acid and DNA base pairs, respectively. The randomly mutated genes were cloned into the vector pET26b(+). Since the *Escherichia coli* strain BL21(DE3) generally has low transformation rates we used the strain BL21 StarTM(DE3), which has a transformation efficiency of over 108 and a better mRNA stability that improves protein yield^37^,^38^,^39^. The *E. coli* cells expressing native AIM-1 are not viable in the presence of >128 μg/mL cefoxitin, and consequently a large library of mutants would need to be generated to find mutants that can survive under these conditions. Since the initial libraries were not large, cefoxitin was set to lower concentrations until a significant number of variants for secondary screening was obtained. The same approach was used in the subsequent three rounds of evolution. However, despite this rather weak to neutral evolutionary pressure the most viable mutants in each round were able to survive in the presence of cefoxitin concentrations well above the MIC (see below). The final (5^th^) round was then carried out with a large library generated with parents that were the best of the previous rounds (some of which had MICs above 128 μg/mL).

The details of the five rounds of evolution are summarised in Table 3. The first round library constructs were transformed into BL21 StarTM(DE3) competent cells and plated on MH agar plates, containing 50 μg/mL kanamycin (vector resistance marker), 50 μg/mL cefoxitin (selection resistance marker) and 50 μM IPTG (to ensure low expression levels). The first round library consisted approximately of 4,000 colonies, which is estimated from a control plate (without the selection resistance marker) of the same batch. About 100 of the colonies survived after an overnight incubation at 37 °C. A secondary selection was then carried out by cultivating these colonies in 96-well plates in LB medium that contained an increased concentration of the substrate (100 μg/mL cefoxitin) while maintaining the concentration of the inducer IPTG at 50 μM. The most viable ten colonies, as judged by the optical density of the cultures at 595 nm (OD595), were used for sequencing to map the mutations; only one of them was a false positive, *i.e*. the wild-type form of AIM-1. The remaining clones contained between one to three mutations (Table 4). These nine first round mutants were the starting point for the second round of library generation, using the same approach as discussed above.

The library after the second iteration contained ~16,000 and ~100 colonies following the primary and secondary selections with 60 μg/mL and 120 μg/mL of cefoxitin, respectively (Table 3). Again, the most viable clones (nine) after the secondary screen were selected for the third round library generation. Of these nine clones, one was a mutant that already appeared in the first round (#2–5 = #1–4), however with the addition of three silent mutations (Table 4). Still, the selected clones again contained between one to three mutations. The third-round library consisted of approximately 33,000 and 200 colonies after selections using 75 μg/mL (primary, on agar plates) and 150 μg/mL cefoxitin (secondary, in liquid media), respectively. A total of 19 mutants from the third library were sequenced; ten of these mutants were already observed in the first and second round libraries. Among the nine novel mutants identified two replacements are particularly prevalent, F114L and F230Y (Table 4). The library size after the fourth round was only ~10,000 for the primary (100 μg/mL cefoxitin) and ~200 (200 μg/mL cefoxitin) for the secondary screen (Table 3). Eleven novel mutants were identified, again with a preference for mutations F114L and, to a smaller extent, F230Y. Some clones contained as many as four mutations (Table 4).

In the final (fifth) round the mutant library was generated using the eStEP method. Genes selected from mutants of the previous four rounds were used to generate a library consisting of ~242,000 and ~300 clones after primary and secondary screening with 125 μg/mL and 250 μg/mL cefoxitin, respectively (Table 3). Of the 300 mutants after the secondary screen several were already present in previous libraries (*e.g*. #3–16, #4–15 and three times #3–11). However, 10 novel mutants, again with a preference for the F114L and F230Y mutations, were observed (Table 4).

It should be noted that a sixth round of evolution was carried out using mutants from the previous five rounds. Over 10,000 colonies were obtained but none of them survived the primary selection at 400 μg/mL cefoxitin. All of the novel mutants identified in round 5, together with three mutants from earlier libraries (and that were still present in the final library), were analysed by MICs assay (Table 5). Similar to the wild-type enzyme all the mutants tested were proficient in hydrolyzing ampicillin (with MIC values all above 1024 μg/mL), and, as expected based on the selection pressure applied, all mutants displayed an increased resistance to cefoxitin (with MIC values ranging from 256 μg/mL to >1024 μg/mL, compared to 128 μg/mL for wild-type AIM-1). However, the mutations had a considerably more diverse effect in presence of the substrate imipenem. While the majority of the mutants are more resistant towards this substrate than the wild-type enzyme (with MIC >1024 μg/mL) two of them are notably more sensitive with MIC values as low as 2 μg/mL (*i.e*. mutant #5–16 and #5–19). In order to obtain more detailed insight into the catalytic effects of the introduced mutations on AIM-1 four mutants (*i.e*. #5–1, #5–2, #5–3 and #5–19) were expressed and purified for further characterization (see below).

The selected mutants have between one to four introduced mutations (Table 5). Two replacements became dominant from the third round of mutagenesis onward, F114L and F230Y (Table 4). Variant #5–2 has only the F114L replacement but is already resistant to all three substrates tested (with MIC values above 1024 μg/mL; Table 5). Other amino acid substitutions, including that at position 230 (as well as S23G, M53T, L55Q, A138V, T154A, P166Q, N247S and S267G), do not seem to improve resistance greatly when compared to position 114 although they appear even after round 5 of mutagenesis. These mutations may thus possibly represent a neutral drift in evolution. On the contrary, mutations in some of these positions may counter the positive effect of the F114L and F230Y mutations, as exampled by variant #5–19, where the additional S23G mutation has a negative impact on the resistance to both cefuroxime and in particular imipenem (compare variants #5–19 and #5–1 in Table 5). Surprisingly, only one of variants observed in the SSM approach (*i.e*. #1–3, T87A) was also obtained via random mutagenesis. This mutant emerged in the first round but was not present in later rounds. Furthermore, the most efficient mutant from the SSM study, F119M, did not appear at all in the directed evolution library. One possible reason for this observation is that the phenylalanine to methionine change requires that two of the three bases of the coding triplet need modification. Given that the mutation rate is low, two changes to a single triplet are rather unlikely.

### Catalytic characterization of selected mutants

From the previous section it has emerged that the mutation F114L has the most significant effect on improving the resistance towards cefoxitin. In order to correlate the recorded MIC values with catalytic parameters, several mutants were selected for further characterization (Table 2). Variants #5–1, #5–2 and #5–3 were selected because they display the highest resistance to each of the three antibiotics tested (with MIC values of 1024 μg/mL or greater; Table 5). Variant #5–19, in contrast, has only mildly improved resistance to cefoxitin but is considerably more sensitive towards imipenem than the wild-type enzyme. As anticipated from the MIC values the catalytic parameters for each of the four mutants do not change dramatically for the reaction with ampicillin; their catalytic efficiencies (*i.e*. their *k*_*cat*_/*K*_*m*_ values) are similar to those of wild-type AIM-1, ranging from 2.2 s^−1^ μM^−1^to 4.2 s^−1^ μM^−1^ (Table 2). The most successful mutant with respect to the substrate ampicillin is F119M, obtained from SSM (note that this mutant has MIC values for each substrate tested >1024 μg/mL).

The kinetic parameters obtained from reactions with cefoxitin are very similar for the three variants #5–1, #5–2 and #5–3; in comparison to the wild-type enzyme their catalytic efficiency is raised from ~0.4 s^−1^ μM^−1^ to ~3 s^−1^ μM^−1^, mainly due to improved interactions with the substrate (*i.e*. lower *K*_*m*_ values). The improved catalytic efficiency is in agreement with the sharply increased MIC values obtained with cultures expressing these mutants (Table 5). The catalytic efficiency of variant #5–19 lies in between that of the other mutants and wild-type AIM-1, also in agreement with the moderate increase in resistance to this antibiotic displayed by cultures expressing this variant. Again, the F119M mutant exceeds the other variants in catalytic performance. For the substrates ampicillin and cefoxitin a good correlation between MIC values and the catalytic efficiencies of the corresponding mutants is observed. However, this is not the case for the substrate imipenem (Tables 2 and 5). The four evolved mutants have catalytic efficiencies ranging from ~2.4 s^−1^ μM^−1^ to 7.6 s^−1^ μM^−1^, with #5–3 being the least efficient. The efficiencies of both the wild-type enzyme and the F119M mutant are similar. However, while cultures expressing variants #5–1, #5–2, #5–3 and F119M each have MIC values above 1024 μg/mL, wild-type AIM-1 has an MIC of 512 μg/mL and variant #5–19 is very sensitive towards imipenem (MIC of 2 μg/mL). The reason for this apparent discrepancy is currently unclear, but it was noted that the yield of expressed variant #5–19 was smaller than that of the other variants. The catalytic characterization of the evolved mutants provides insight into the mechanism of evolution. For variants #5–1, #5–2 and #5–3 a significant improvement in their interaction with the substrate that exerts the selection pressure (*i.e*. cefuroxime) is observed, with *K*_*m*_ values dropping from nearly 300 μM for the wild-type enzyme to ~20 to ~40 μM for the mutants (Table 2). Interestingly, the same trend was also observed for the substrate imipenem (but not ampicillin). Considering that the concentration of selection-inducing β-lactam is likely to be considerably lower in the periplasm than in the surrounding medium a decrease in *K*_*m*_ may be the preferred option rather than evolving mutants with increased *k*_*cat*_ values. This hypothesis is supported by the observation that both the evolved variant #5–19 and in particular the engineered mutant F119M have enhanced *k*_*cat*_ values towards some or all of the substrates when compared to the other variants and the wild-type enzyme, but their MIC values are not better than those of variants #5–1, #5–2 and #5–3. This observed discrepancy in the enhancement of MIC values and catalytic efficiency may be due to impaired metal ion binding of the two mutants when compared to the wild-type enzymes and variants #5–1–#5–3, as was observed for some mutants of the B1-type MBL BcII^30^. However, it appears more likely that due to the significantly increased *K*_*m*_ for cefoxitin both the F119M mutant and #5–19 variant operate at sub-saturated levels in the *in vivo* assays.

### Structure of variant #5–1

In order to gain insight into the effect of the introduced mutations on the structure of AIM-1 variant #5–1 was selected for a crystallographic study. The structure was solved to a resolution of 1.89 Å (Table 6) and has an overall fold that is virtually identical to that of the wild-type enzyme (data not shown). The variant contains the two mutations identified as the most prevalent, *i.e*. F114L and F230L (see above; the third mutation present, L55Q, is not expected to have a significant effect). Since the mutation F114L is the only replacement in variant #5–2 (Tables 2 and 5), leading to a considerable drop in the *K*_*m*_ values of both cefuroxime and imipenem, as well as an increase in the corresponding MIC values, it is plausible to assume that this residue plays a pivotal role in substrate recognition. Surprisingly, the effect on the active site structure is rather minimal (Fig. 4). The coordination environment of the two metal ions in the active site, as well as the metal-metal distance, are virtually unchanged, demonstrating that subtle structural changes in the outer sphere of the catalytic centre are sufficient to profoundly affect the substrate preference of an MBL. The other frequent mutation, F230Y, is located on a loop rather distant from the active site. Based on a comparison of the catalytic parameters of the variants in Table 3 this substitution is not likely to have a significant effect on substrate recognition or catalysis. It thus appears that only minimal changes are required to change the catalytic performance (including substrate selectivity) of AIM-1 (the major differences according to a comparison of the structures of wild-type and mutant AIM-1 are the hydrogen bonding and water molecule arrangements between regions 113–115 and 199–202; Fig. 4). Indeed, the observed minimal crystallographic changes are in agreement with a similar observation reported for the B1-type MBL BcII; in that enzyme the improved activity towards an increased range of substrates could also not be rationalised by crystal structures that provide static snapshots^32^. Instead it is conformational dynamics that is likely to play an essential role in expanding the substrate promiscuity of both enzymes. Protein dynamics has been recognised as a major factor controlling various aspects of enzyme function, including substrate binding^40^,^41^. For BcII a recent study using NMR indeed demonstrated that successful evolution is epistatic and increases protein loop dynamics on a timescale related to catalytic turnover rates (*i.e*. micro- to milliseconds)^29^, and it is very likely that in variant #5–1 of AIM-1 increased loop dynamics are also introduced by the three mutations.

## Conclusions

**Figure 1:**
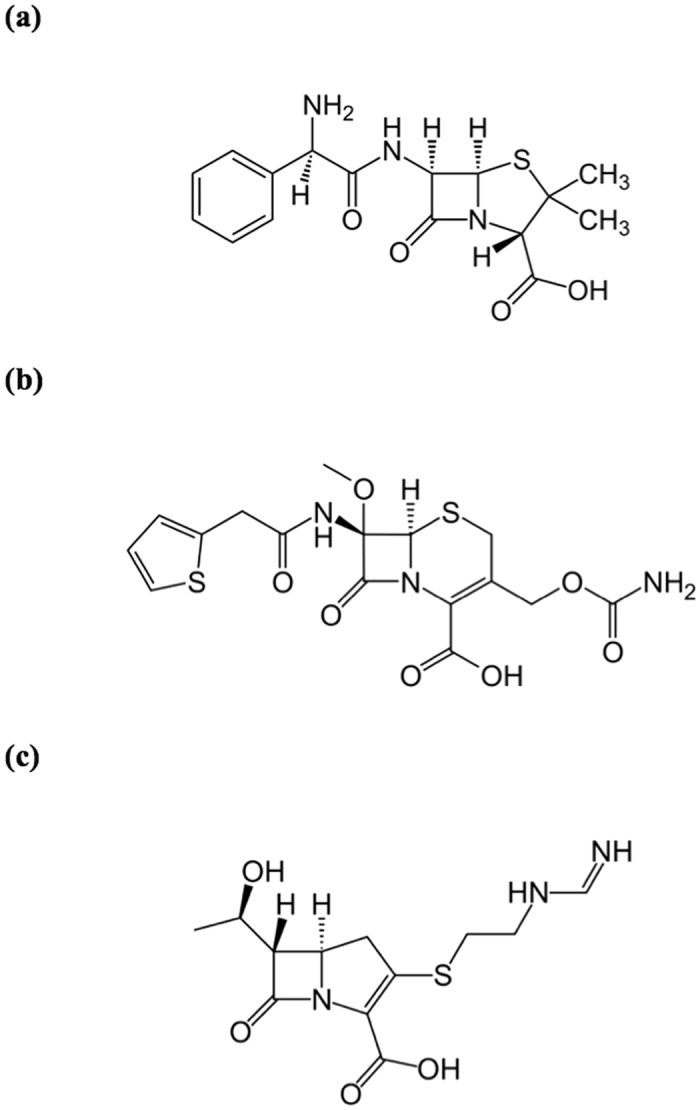
Structure of three representative β-lactam compounds used in this study. (**a**) Ampicillin, a penicillin; (**b**) cefoxitin, a cephalosporin; (**c**) imipenem, a carbapenem.

AIM-1 is one of the most efficient MBLs with a particular preference for penicillin-related compounds but also carbapenems (Fig. 1; Table 2)^17^,^18^. The enzyme is less effective towards cephalosporins. However, with a substrate preference similar to that of NDM-1 the enzyme has the potential to become a significant threat to health care. MBLs pose a significant problem because no clinically useful inhibitors are currently available^42^, but also because they appear to be able to easily adapt to new substrates (*i.e*. β-lactam antibiotics). In this study we intended to probe the ease with which such enzymes may adapt. For that purpose two complementary strategies were employed.

**Figure 2:**
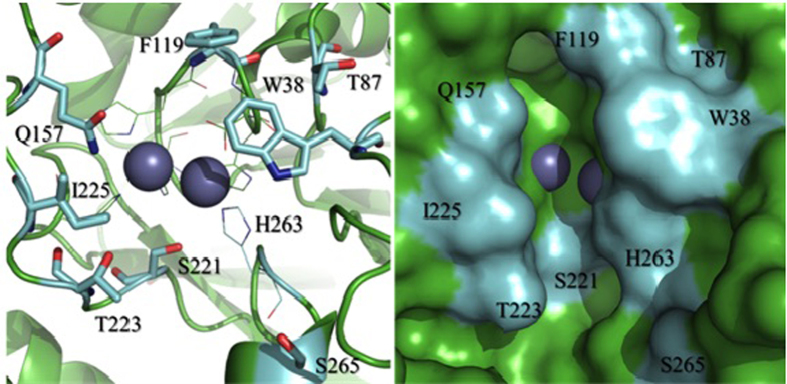
Surface active site residues in AIM-1. (**a**) A stick presentation; (**b**) a surface view. The diagrams were generated using the crystal structure of wild-type AIM-1 (PDB code: 4AWY)^34^.

**Figure 3:**
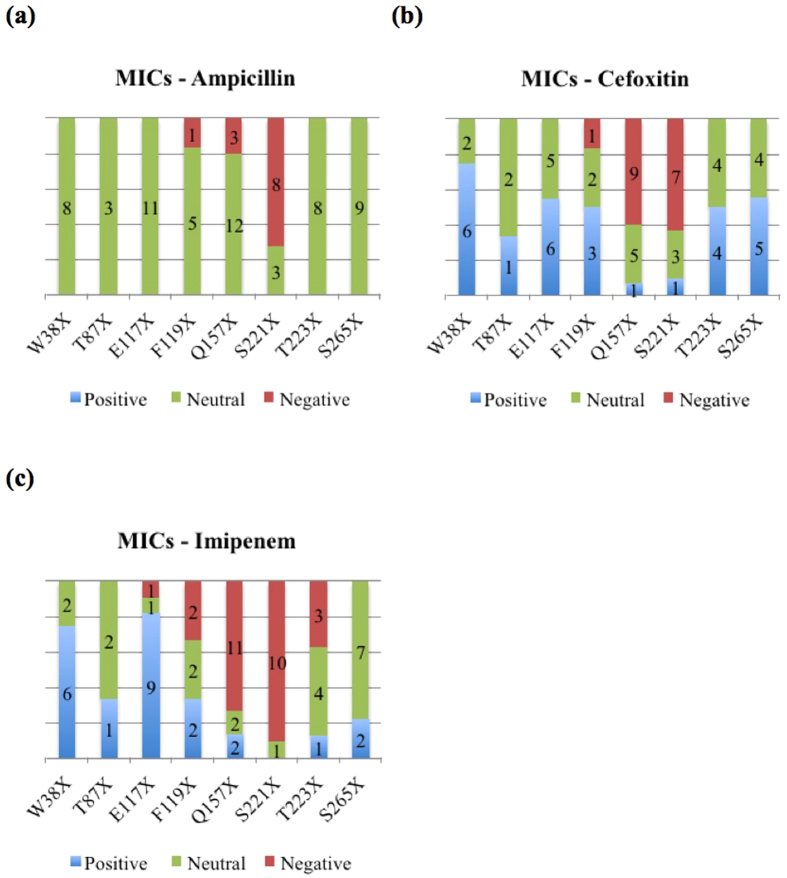
MIC results of (**a**) ampicillin, (**b**) cefoxitin, and (**c**) imipenem. The bars illustrate the substitution tolerance/sensitivity of selected amino acids in the vicinity of the active site of AIM-1, probed by SSM. Numbers of distinct mutants are shown in the bars and the colours represent mutants that have either improved, unaltered or reduced activity toward a specific substrate.

Firstly, guided by the crystal structure of AIM-1 (Fig. 2) a number of residues in the vicinity of the catalytic centre were predicted to play an important role in substrate recognition. Indeed, using SSM we could demonstrate that through single mutations the preference of substrates can be greatly altered (Fig. 3). Of particular relevance was the mutation F119M, which raised the efficiency of AIM-1 towards the cephalosporin substrate cefoxitin ten-fold (Table 2).

**Figure 4:**
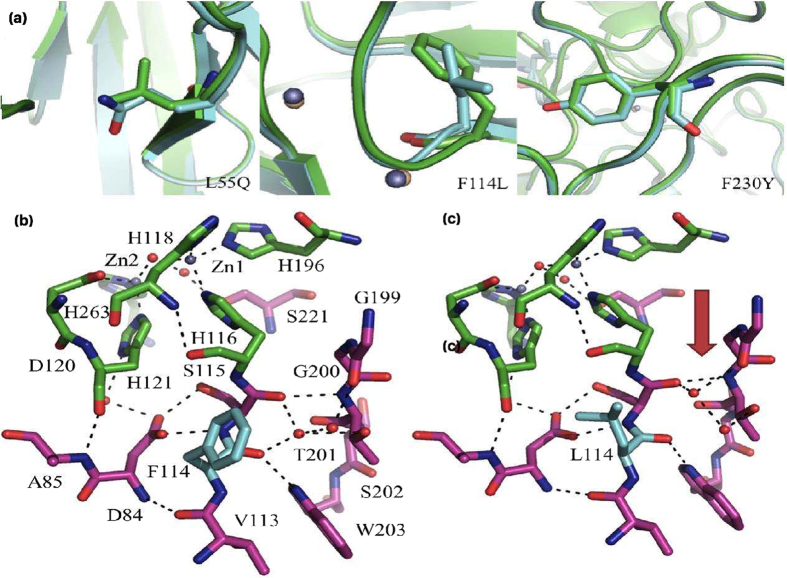
Comparison of the structures of mutant #5−1 and wild type AIM-1^34^. In (**a**) the illustrations focus on the three introduced mutations *i.e*. L55Q, F114L and F230Y. In (**b**,**c**) the active sites of the wild-type and mutant AIM-1, respectively, are shown. First and second coordination sphere residues are shown in green and magenta, respectively. The only amino acid variation is the active site (*i.e*. F114L) is shown in cyan. The only significant variation is the orientation of two water molecules and associated hydrogen bonds, indicated by the red arrow.

Secondly, several rounds of *in vitro* evolution were carried out in order to simulate a pathogen’s response towards increasing concentrations of an antibiotic. Cefoxitin was selected as substrate since wild-type AIM-1 displays a considerable bias against cephalosporins. Within five rounds of evolution a series of variants were obtained with vastly improved resistance towards that substrate (Table 5). Furthermore, similar to the SSM approach it emerged that only a single mutation, *i.e*. F114L, is necessary to induce a significant improvement of the bacterial host’s resistance towards a broad spectrum of antibiotics (with MIC values >1024 μg/mL), thus illustrating how easily a potential “super bug” may be generated. This mutation has a minimal effect on the structure of the enzyme (Fig. 4), thus demonstrating that MBLs have the capacity to fine-tune their substrate preference without compromising their catalytic efficiency.

This study also demonstrates that MBLs can evolve resistance to new substrates by mutating residues that are not easily predictable from an available crystal structure. Hydrophobic side chains such as F114 or F230 were not identified as residues that may play an important role in substrate recognition from a structural analysis but from an *in vitro* evolution experiment. The identification of several relevant hydrophobic residues in substrate interactions (in particular F114, F119 and F230) is in agreement with similar observations reported for the B1-type MBLs BcII, SPM-1 and IMP, where residues G262 (BcII and SPM-1) and V67 (IMP) play essential roles in substrate recognition^25^,^30^,^43^. While the structural origins for the contributions of these hydrophobic residues to substrate binding and hydrolysis remain to be elucidated their identification indicates that MBLs have a diverse array of possibilities to adapt to new selection pressures, thus compounding their threat to health care. It is thus essential to devise alternative approaches to combat the efficiency of such enzymes. It appears that the one factor common to all these enzymes is their reliance on metal ions for their function. Indeed, in a recent study Vila and coworkers have demonstrated that the relationship between protein stability, metal ion binding and catalytic activity is very important for the function of MBLs, in agreement with an earlier study on a distantly related organophosphate-degrading metallohydrolase from *Agrobacterium radiobacter*^30^,^44^. In particular, these studies illustrate that the optimisation of metal ion binding in these enzymes may play an important role in adapting their catalytic efficiencies. This interpretation is further supported by a recent study with AIM-1, where an analysis of the metal ion binding affinity indicated that in this enzyme two Zn(II) ions bind with comparable affinity (*K*_*d*_~170 nM), whereas the same active site displays a very tight affinity for one Co(II) ion (*K*_*d*_~7 nM), with a second Co(II) binding much weaker (*K*_*d*_~2 μM)^18^. Since the Zn(II) derivative of AIM-1 is considerably more reactive than its Co(II) counterpart, variations in metal ion binding may indeed correlate with reactivity and fitness. Hence, an efficient strategy to incapacitate these enzymes ought to target their metal ion binding interactions. Efforts towards this goal are currently in progress.

## Methods

### Protein cloning, expression and purification

The codon-optimized DNA sequence encoding AIM-1 was purchased from DNA2.0 Inc. (Menlo Park, CA, USA). The gene and its variants were cloned into a site flanked by the NdeI and EcoRI restriction sites in the pET26b(+) vector (Novagen) for recombinant expression. Instead of using the pelB leader peptide, AIM-1 uses its own leading peptide to the periplasm of *E. coli*. The expressed enzyme was purified using a Q Sepharose anion-exchange column followed by gel filtration on a Sephadex G75 column. The anion exchange column was equilibrated with 50 mM Tris-HCL, 150 μM ZnCl_2_ and 10% glycerol, at pH 7.6 (except for the S221E mutant of AIM-1, where the pH was lowered to 6.5). The protein was eluted by increasing the NaCl concentration gradually to 1 M. The gel filtration column was equilibrated with the same buffer, and the eluted protein solution was concentrated to ~1 mg/mL and stored at −20 °C (in 10% glycerol). The concentration of purified protein was measured using an ND-1000 spectrophotometer system (NanoDrop Technologies, Wilmington, DE, USA).

### Protein crystallization

For crystallization the protein was transferred into 20 mM Tris-HCL, 150 μM ZnCl_2_, 10% glycerol, pH 7.6, and further concentrated (~20 mg/mL). The sitting-drop vapour diffusion method was employed at 4 °C in 96-well plates (Art Robbins Instruments, Sunnyvale, CA, USA). Every drop contained 0.5 μL of the protein sample and 0.5 μL of the screen solution (Hampton crystal screens). A total of 495 conditions were tested using a robot dispensing system. The most suitable condition was 0.2 M zinc acetate in 0.1 M sodium cacodylate, *pH* 5.0, with 5% to 18% (w/v) PEG 8000.

### Data collection, structure determination, refinement and validation

X-ray diffraction data were collected on the Australian Synchrotron MX2 (Melbourne), using an ADSC Quantum 315r detector. The structure of native AIM-1^34^ was used as a search model to solve the structure by molecular replacement. Iterative manual adjustments using REFMAC5 (CCP4)^45^,^46^,^47^ and Coot^48^ allowed the refinement of the model to converge to a final R_work_ and R_free_ of 17.5 and 19.9%, respectively (Table 6). The stereochemistry of the structures was checked with Sfcheck (CCP4)^48^. The main-chain torsion angles of the model were 97.4% with 0 outliers.

### Site-saturation mutagenesis

Site-saturation mutagenesis (SSM) primers were designed using NNK that cover 20 different amino acids. Flanking the mutation-inducing codons are at least 12 bases on either side, resulting in primers of 27 to 33 base pairs in length. All primers were purchased from Geneworks (Thebarton, SA, Australia) or IDT DNA (Coralville, IA, USA). Most of the active site residues that are not directly involved in metal ion binding were subjected to SSM experiments, including W38, T87, E117, F119, Q157, S221, T223, I225 and S265 (Fig. 2). SSM was carried as described elsewhere^49^.

### Directed evolution

Directed evolution involved two major steps, *i.e*. library generation and selection. For the generation of the library the gene of the target protein was subjected to mutagenesis either via StEP, ePCR or StEP-ePCR^50^, and as implemented by Stevenson and colleagues^51^. The forward and reverse primer sequences were 5′-GGA GAT ATA CAT ATG AAA CGT CGC TTC ACC-3′ and 5′-AAG CTT GAA TTC TTA CGG GCG AGC ACC GCT-3, respectively. The resulting PCR products were cloned into the pET26b(+) vector, and the constructs were then transferred into BL21 StarTM(DE3) (Invitrogen, California, USA). Subsequently, the transformed cells were grown on Mueller-Hinton agar plates, supplemented with 50 μg/mL kanamycin and 50–125 μg/mL cefoxitin for selection. To ensure a low expression level a final concentration of 50 μM isopropyl-β-D-1- thiogalactopyranoside (IPTG) (Solon, OH, USA) was used in all selection media. Secondary screening was performed on 96-well plates to find the best-performing mutants and the genes were pooled for subsequent rounds of directed evolution.

### Minimum inhibition concentration (MIC) assay

MIC values were determined following a published procedure^52^. The range of antibiotic concentrations was diluted from a 1 mg/L stock solution as required. LB or MH media plus 50 μg/mL kanamycin were used as the base media, and 96-well plates were used to set up the target antibiotic concentration gradients. Each plate carried eight samples including a positive and a negative control. Each sample was in a row on the plate with 12 different concentrations (up to 1024 μg/ml) of an antibiotic. Samples were pre-cultured overnight then 1 μL of each sample was added to each well. The assays were carried out in triplicates to ensure consistency of the outcomes.

### Enzymatic studies

A Cary 50 Bio Varian UV-Vis spectrophotometer was used to measure catalytic rates at wavelengths ranging from 235 to 405 nm depending on the substrates. Quartz cuvettes (1 cm path length) were used and the assays were performed in 50 mM HEPES buffer, pH 7.6, containing 50 μM ZnCl_2_, at 30 °C. Measurements were carried out in triplicates. The optimal wavelengths (and corresponding extinction coefficients) of the substrates used are: ampicillin, 235 nm (ε = −820 M^−1^ cm^−1^); cefoxitin, 260 nm (ε = −7700 M^−1^ cm^−1^); imipenem, 300 nm (ε = −9000 M^−1^ cm^−1^). The experimental data were analysed using the Michaelis-Menten equation (equation 1)^53^.


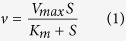


## Additional Information

**How to cite this article**: Hou, C.-F. D. *et al*. Insights into an evolutionary strategy leading to antibiotic resistance. *Sci. Rep.* **7**, 40357; doi: 10.1038/srep40357 (2017).

**Publisher's note:** Springer Nature remains neutral with regard to jurisdictional claims in published maps and institutional affiliations.

## Figures and Tables

**Table 1:** List of SSM mutants.

W38X	T87X	E117X	F119X	Q157X	S221X	T223X	S265X
A	A	A	A	A	A	A	A
L	S	L	L	I	L	I	M
F	G	F	M	L	W	S	T
V		W	W	M	V	G	N
S		Y	S	W	Q	P	Q
T		V	Q	Y	C	R	C
Q		T		V	G	K	G
C		C		S	R	E	R
R		G		T	K		D
		R		G	D		
		D		P	E		
				R			
				H			
				D			
				E			

**Table 2:** Catalytic parameters of wild-type AIM-1 and selected mutants. Parameters were determined from reactions with three different substrates.

	Ampicillin			Cefoxitin			Imipenem		
	*k*_*cat*_ (s^−1^)	*K*_*M*_ (μM)	*k*_cat_/*K*_*M*_ (s^−1^ μM^−1^)	*k*_cat_ (s^−1^)	*K*_*M*_ (μM)	*k*_cat_/*K*_*M*_ (s^−1^ μM^−1^)	*k*_cat_ (s^−1^)	*K*_*M*_ (μM)	*k*_cat_/*K*_*M*_ (s^−1^μM^−1^)
AIM-1	410 ± 30	110 ± 21	3.8	117 ± 9	283 ± 37	0.4	1405 ± 242	289 ± 66	5.0
W38C	131 ± 11	92 ± 21	1.5	13 ± 2	12 ± 3	1.2	254 ± 41	157 ± 30	1.6
F119M	2327 ± 754	388 ± 163	6.2	122 ± 19	30 ± 11	4.3	1240 ± 400	279 ± 102	4.6
S221A	251 ± 62	434 ± 190	0.6	54 ± 3	19 ± 2	3.0	23 ± 4	70 ± 12	0.3
S221E	0.4 ± 0.2	57000 ± 25000	~0	~0	1018 ± 58	~0	ND	ND	ND
#5–1	382 ± 3	92 ± 7	4.2	60 ± 2	17.6 ± 2	3.5	82.9 ± 7	11 ± 1	7.6
#5–2	481 ± 35	206 ± 49	2.4	79 ± 7	28 ± 2	2.8	141 ± 20	40 ± 5	3.5
#5–3	440 ± 17	140 ± 12	3.2	93 ± 5	36 ± 5	2.6	196 ± 49	87 ± 34	2.4
#5–19	2335 ± 361	1065 ± 216	2.2	352 ± 44	237 ± 44	1.5	40 ± 3	13 ± 2	3.1

**Table 3:** Directed evolution of AIM-1 using cefoxitin for selection pressure.

Evolution Rounds	Methods	Cefoxitin (Agar/Liquid)	Library Size (*n*) (Primary/Secondary)
Round 1	ePCR	50/100 μg/mL	4,000/100
Round 2	ePCR	60/120 μg/mL	16,000/100
Round 3	ePCR	75/150 μg/mL	33,000/200
Round 4	ePCR	100/200 μg/mL	10,000/200
Round 5	eStEP	125/250 μg/mL	242,000/300

**Table 4:** Directed evolution of AIM-1 over 5 rounds.

Round 1	S18	S23	D33	G36	P51	M53	L55	Y63	T87	R99	L101	F114	Q129	A138	P141	I143	T154	P166	N169	T172
#1–1										H		L								
#1–2												S	R							
#1–3								H	A											
#1–4																				
#1–5						T									L					
#1–6																		Q		
#1–7																				
#1–8			E		Q															
#1–9																				
Round 2																				
#2–1				D																
#2–2			E																	
#2–3											S							Q		
#2–4		G																		
#2–5																				
#2–6												S			L					
#2–8																	A			
#2–9										H		L								
Round 3																				
#3–1																				
#3–2																				A
#3–3												I								
#3–7										H		L		V						
#3–8																				
#3–11												L								
#3–13							Q					L								
#3–16												T					A			
#3–19										H		L								
Round 4																				
#4–1												T					A			
#4–2												L								
#4–5						T				H		L								
#4–7							Q					L					A			
#4–8										H		L								
#4–9																				
#4–10												L		V						
#4–12	P											L								
#4–15							Q					L								
#4–16												L								
#4–19												I							D	
Round 5																				
#5–1							Q					L								
#5–2												L								
#5–3		G										L								
#5–12												L		V						
#5–14						T														
#5–16												L				T				
#5–17		G										L						Q		
#5–18												L					A			
#5–19		G										L								
#5–20												L								
Round 1	D176	V183	S207	A224	F230	D235	A237	L242	N247	T248	S267	I273	Q292	R295	R297	K300	A307			
#1–1																				
#1–2																				
#1–3																				
#1–4														H						
#1–5					Y															
#1–6																				
#1–7						V														
#1–8												V								
#1–9		I																		
Round 2																				
#2–1	G													H						
#2–2												V								
#2–3																				
#2–4					Y						G									
#2–5															H					
#2–6																				
#2–8																				
#2–9					Y															
Round 3																				
#3–1					Y		S													
#3–2					Y															
#3–3		I																		
#3–7																				
#3–8				P	Y															
#3–11					Y															
#3–13									S											
#3–16																				
#3–19																E				
Round 4																				
#4–1																	V			
#4–2									G											
#4–5					Y															
#4–7					Y															
#4–8								Q												
#4–9					Y						G									
#4–10									S											
#4–12					Y					A										
#4–15																				
#4–16													R			E				
#4–19																				
Round 5																				
#5–1					Y															
#5–2																				
#5–3					Y				S											
#5–12																				
#5–14					Y															
#5–16					Y						G									
#5–17									S											
#5–18																				
#5–19					Y															
#5–20			I																	

**Table 5:** MIC values (μg/mL) of engineered mutants. Mutants #3–11, #3–16 and #4–15 survived to the 5^th^ round of evolution.

Mutants	Ampicillin	Cefoxitin	Imipenem	Mutations
Wild type	>1024	128	512	
#3–11	>1024	256	512	F114L F230Y
#3–16	>1024	1024	>1024	F114T T154A
#4–15	>1024	1024	>1024	L55Q F114L
#5–1	>1024	>1024	>1024	L55Q F114L F230Y
#5–2	>1024	1024	>1024	F114L
#5–3	>1024	1024	>1024	S23G F114L F230Y N247S
#5–12	>1024	512	1024	F114L A138V
#5–16	>1024	256	4	F114L I143T F230Y S267G
#5–17	>1024	512	512	S23G F114L P166Q N247S
#5–18	>1024	256	128	F114L T154A
#5–19	>1024	256	2	S23G F114L F230Y
#5–20	>1024	1024	>1024	F114L S207I

**Table 6:** Data collection, phasing and refinement statistics.

Statistic	#5–1
**Data collection**	
Space group	P6_1_22
**Cell dimensions**	
a, b, c (Å)	76.45, 76.45, 240.27
Resolution (Å)	66.21–1.89
Reflections (*n*)	34233
*R*_sym_ or *R*_merge_	99.7 (98.1)
*I*/*σI*	25 (6.9)
Completeness (%)	99.7 (98.1)
Redundancy	12.7 (7.2)
**Refinement**	
Resolution (Å)	1.89
No. reflections	32,428
*R*_work_/*R*_free_	0.175/0.200
Residues (*n*)	
Protein	271
Zinc ion	2
Water	214
*B* factors	
Protein	17.929
Ligand/ion	25.524
Water	23.007
**R**.**m**.**s**. **deviations**	
Bond lengths (Å)	0.018
Bond angles (deg)	1.812
pH	5.0
PDB ID	4P62

## References

[b1] Costerton J. W., Stewart P. S. & Greenberg E. P. Bacterial biofilms: a common cause of persistent infections. Science 284, 1318–1322 (1999). 10334980 10.1126/science.284.5418.1318

[b2] De Spiegeleer P., Sermon J., Vanoirbeek K., Aertsen A. & Michiels C. W. Role of porins in sensitivity of *Escherichia coli* to antibacterial activity of the lactoperoxidase enzyme system. Appl Environ MicroBiol. 71, 3512–3518 (2005). 16000755 10.1128/AEM.71.7.3512-3518.2005 PMC1169026

[b3] Zapun A., Contreras-Martel C. & Vernet T. Penicillin-binding proteins and β-lactam resistance. FEMS Microbiol Rev. 32, 361–385 (2008). 18248419 10.1111/j.1574-6976.2007.00095.x

[b4] Drawz S. M. & Bonomo R. A. Three decades of β-lactamase inhibitors. Clin Microbiol Rev. 23, 160–201 (2010). 20065329 10.1128/CMR.00037-09 PMC2806661

[b5] Crowder M. W., Spencer J. & Vila & Metallo-β-lactamases: novel weaponry for antibiotic resistance in bacteria. Acc Chem Res. 39, 721–728 (2006). 17042472 10.1021/ar0400241

[b6] Mitić N. . Catalytic mechanisms of metallohydrolases containing two metal ions. Adv Protein Chem Struct Biol. 97, 49–81 (2014). 25458355 10.1016/bs.apcsb.2014.07.002

[b7] Phelan E. K. . Metallo-β-Lactamases: A Major Threat to Human Health. Am J Mol Biol. 04(89), 43011 (2014).

[b8] Payne D. J. Metallo-β-lactamases - a new therapeutic challenge. J Med MicroBiol. 39, 93–99 (1993). 8345513 10.1099/00222615-39-2-93

[b9] Wang Z., Fast W. & Benkovic S. J. On the mechanism of the metallo-β-lactamase from *Bacteroides fragilis*. Biochemistry 38, 10013–10023 (1999). 10433708 10.1021/bi990356r

[b10] Ambler R. P. The structure of β-lactamases. Philos Trans R Soc Lond B Biol Sci. 289, 321–331 (1980). 6109327 10.1098/rstb.1980.0049

[b11] Garau G. . Standard numbering scheme for class B β-lactamases. Antimicrob Agents Chemother. 45, 660–663 (2001). 11181339 10.1128/AAC.45.3.660-663.2001 PMC90352

[b12] Karsisiotis A. I., Damblon C. F. & Roberts G. C. A variety of roles for versatile zinc in metallo-β-lactamases. Metallomics. 6, 1181–1197 (2014). 24696003 10.1039/c4mt00066h

[b13] Valladares M. . Zn(II) dependence of the *Aeromonas hydrophila* AE036 metallo-β-lactamase activity and stability. Biochemistry. 36, 11534–11541 (1997). 9298974 10.1021/bi971056h

[b14] Garau G., Bebrone C., Anne C. & Galleni M. A metallo-β-lactamase enzyme in action: crystal structures of the mono zinc carbapenemase CphA and its complex with biapenem. J Mol Biol. 345, 785–95 (2005). 15588826 10.1016/j.jmb.2004.10.070

[b15] Vella P. & Mitic N. . Identification and characterization of an unusual metallo-β-lactamase from *Serratia proteamaculans*. J Biol Inorg Chem. 18, 855–863 (2013). 23982345 10.1007/s00775-013-1035-z

[b16] Hou C. F. D., Phelan E. K., Miraula M. & Ollis D. L. Unusual metallo-β-lactamases may constitute a new subgroup in this family of enzymes. Am J Mol Biol. 4, 11–15 (2014).

[b17] Yong D. . Genetic and biochemical characterization of an acquired subgroup B3 metallo-β-lactamase gene, *bla*_*AIM-1*_, and its unique genetic context in *Pseudomonas aeruginosa* from Australia. Antimicrob Agents Chemother. 56, 6154–6159 (2012). 22985886 10.1128/AAC.05654-11 PMC3497169

[b18] Selleck . AIM-1: An antibiotic-degrading metallohydrolase that displays mechanistic flexibility. Chem Eur J., doi: 10.1002/chem.201602762 (2016). 27778387

[b19] Wachino J., Yoshida H. & Yamane K. SMB-1, a novel subclass B3 metallo-β-lactamase, associated with ISCR1 and a class 1 integron, from a carbapenem-resistant *Serratia marcescens* clinical isolate. Antimicrob Agents. 55, 5143–5149 (2011). 10.1128/AAC.05045-11 PMC3195065 21876060

[b20] Wachino J., Mori S., Yamagata Y., Arakawa Y. & Shibayama K. Crystallization and preliminary X-ray analysis of the subclass B3 metallo-β-lactamase SMB-1 that confers carbapenem resistance. Acta Crystallogr Sect F Struct Biol Cryst Commun. F68, 343–346 (2012). 10.1107/S1744309112004691 PMC3310548 22442240

[b21] Miraula M. B., Selleck C., Schenk G. & Mitić N. Identification and preliminary characterization of novel B3-type metallo-β-lactamases. Am J Mol Biol. 3, 198–203 (2013).

[b22] Miraula M., Whitaker J. J., Schenk G. & Mitić N. β-Lactam antibiotic-degrading enzymes from non-pathogenic marine organisms: a potential threat to human health. J Biol Inorg Chem. 20, 639–651 (2015). 25773168 10.1007/s00775-015-1250-x

[b23] Miraula M., Schenk G. & Mitić N. Promiscuous metallo-β-lactamases: MIM-1 and MIM-2 may play an essential role in quorum sensing networks. J Inorg Biochem. 162, 366–375 (2016). 26775612 10.1016/j.jinorgbio.2015.12.014

[b24] Toleman M. A., Simm A. M. & Murphy T. A. Molecular characterization of SPM-1, a novel metallo-β-lactamase isolated in Latin America: report from the SENTRY antimicrobial surveillance programme. J Antimicrob Chemother. 50, 673–679 (2002). 12407123 10.1093/jac/dkf210

[b25] LaCuran A. E. . Elucidating the Role of Residue 67 in IMP-Type Metallo-β-Lactamase Evolution. J Antimicrob Chemother. 59, 7299–7307 (2015). 10.1128/AAC.01651-15 PMC4649200 26369960

[b26] Pegg K. M. . Understanding the determinants of substrate specificity in IMP family metallo-β-lactamases: the importance of residue 262. Prot Sci. 23, 1451–1460 (2014). 10.1002/pro.2530 PMC4287004 25131397

[b27] Oelschlaeger P., Mayo S. L. & Pleiss J. Impact of remote mutations on metallo-β-lactamase substrate specificity: implications for the evolution of antibiotic resistance. Prot Sci. 14, 765–774 (2005). 10.1110/ps.041093405 PMC2279297 15722450

[b28] Materon I. C., Beharry Z., Huang W., Perez C. & Palzkill T. Analysis of the context dependent sequence requirements of active site residues in the metallo-β-lactamase IMP-1. J Mol Biol. 344, 653–663 (2004). 15533435 10.1016/j.jmb.2004.09.074

[b29] González M. M., Abriata L. A., Tomatis P. E. & Vila A. J. Optimization of Conformational Dynamics in an Epistatic Evolutionary Trajectory. Mol Biol Evol. 3(7), 1768–1776 (2016). 10.1093/molbev/msw052 PMC5854100 26983555

[b30] Meini M. R., Tomatis P. E., Weinreich D. M. & Vila A. J. Quantitative description of a protein fitness landscape based on molecular features. Mol Biol Evol. 32(7), 1774–1787 (2015). 25767204 10.1093/molbev/msv059 PMC4476158

[b31] Tomatis P. E., Rasia R. M. & Segovia L. Mimicking natural evolution in metallo-β-lactamases through second-shell ligand mutations. Proc Natl Acad Sci USA 102(39), 13761–13766 (2005). 16172409 10.1073/pnas.0503495102 PMC1236536

[b32] Tomatis P. E., Fabiane S. M. & Simona F. Adaptive protein evolution grants organismal fitness by improving catalysis and flexibility. Proc Natl Acad Sci USA 05(52), 20605–20610 (2008). 10.1073/pnas.0807989106 PMC2634896 19098096

[b33] Sun S., Zhang W., Mannervik B. & Andersson D. I. Evolution of broad spectrum β-lactam resistance in an engineered metallo-β-lactamase. J Biol Chem. 288(4), 2314–2324 (2013). 23209299 10.1074/jbc.M112.430199 PMC3554903

[b34] Leiros H. K. . Antimicrob Agents Chemother 56, 4341–4353 (2012). 22664968 10.1128/AAC.00448-12 PMC3421596

[b35] Mercuri P. S., Garcia-Saez I. & Vriendt D. K. Probing the specificity of the subclass B3 FEZ-1 metallo-β-lactamase by site-directed mutagenesis. J Biol Chem. 6(279), 32, 33630–33638 (2004). 10.1074/jbc.M403671200 15159411

[b36] Spencer J., Read J., Sessions R. B. & Howell S. Antibiotic recognition by binuclear metallo-β-lactamases revealed by X-ray Crystallography. J Am Chem Soc 19(127), 41, 14439–14444 (2005). 10.1021/ja0536062 16218639

[b37] Grunberg-Manago M. Messenger RNA Stability and its role in control of gene expression in bacteria and phages. Annu Rev Genet. 33, 193–227 (1999). 10690408 10.1146/annurev.genet.33.1.193

[b38] Kido M., Yamanaka K., Mitani T., Niki H., Ogura T. & Hiraga S. RNase E polypeptides lacking a carboxyl-terminal half suppress a mukB mutation in *Escherichia coli*. J Bacteriol. 178, 3917–3925 (1996). 8682798 10.1128/jb.178.13.3917-3925.1996 PMC232654

[b39] Lopez P. J., Marchand I., Joyce S. A. & Dreyfus M. The C-terminal half of RNase E, which organizes the *Escherichia coli* degradosome, participates in mRNA degradation but not rRNA processing *in vivo*. Mol Microbiol. 33, 188–199 (1999). 10411735 10.1046/j.1365-2958.1999.01465.x

[b40] Tokuriki N. & Tawfik D. S. Protein dynamism and evolvability. Science 324(5924), 203–207 (2009). 19359577 10.1126/science.1169375

[b41] Jackson C. J. . Conformational sampling, catalysis, and evolution of the bacterial phosphotriesterase. Proc Natl Acad Sci USA 106, 21631–21636 (2009). 19966226 10.1073/pnas.0907548106 PMC2799856

[b42] McGeary R. P., Schenk G. & Guddat L. W. The applications of binuclear metallohydrolases in medicine: recent advances in the design and development of novel drug leads for purple acid phosphatases, metallo-β-lactamases and arginases. Eur J Med Chem. 76, 132–44 (2014). 24583353 10.1016/j.ejmech.2014.02.008

[b43] González L. J., Moreno D. M., Bonomo R. A. & Vila A. J. Host-specific enzyme-substrate interactions in SPM-1 metallo-β-lactamase are modulated by second sphere residues. PLoS Pathog. 10(1), e1003817 (2014). 24391494 10.1371/journal.ppat.1003817 PMC3879351

[b44] Foo J. L., Jackson C. J., Carr P. D., Kim H. K. & Schenk G. Mutation of outer-shell residues modulates metal ion co-ordination strength in a metalloenzyme. Biochem J. 15, (429), 2, 313–321 (2010). 10.1042/BJ20100233 20459397

[b45] Murshudov G. N., Vagin A. A. & Dodson E. J. Refinement of macromolecular structures by the maximum-likelihood method. Acta Crystallogr Sect D Biol Cryst. 53(3), 240–255 (1997). 15299926 10.1107/S0907444996012255

[b46] Murshudov G. N., Skubák P. & Lebedev A. A. REFMAC5 for the refinement of macromolecular crystal structures. Acta Crystallogr D Biol Crystallogr. 67(4), 355–367 (2011). 21460454 10.1107/S0907444911001314 PMC3069751

[b47] Emsley P., Lohkamp B., Scott W. G. & Cowtan K. Features and development of Coot. Acta Crystallogr D Biol Crystallogr. 66(04), 486–501 (2010). 20383002 10.1107/S0907444910007493 PMC2852313

[b48] Vaguine A. A., Richelle J. & Wodak S. J. SFCHECK: a unified set of procedures for evaluating the quality of macromolecular structure-factor data and their agreement with the atomic model. Acta Crystallogr D Biol Crystallogr. 55, 191–205 (1999). 10089410 10.1107/S0907444998006684

[b49] Ng T. K., Gahan L. R., Schenk G. & Ollis D. L. Altering the substrate specificity of methyl parathion hydrolase with directed evolution. Arch Biochem Biophys. 1(573), 59–68 (2015). 10.1016/j.abb.2015.03.012 25797441

[b50] Zhao H., Giver L., Shao Z. & Affholter J. A. Molecular evolution by staggered extension process (StEP) *in vitro* recombination. Nat Biotechnol. 16(3), 258–61 (1998). 9528005 10.1038/nbt0398-258

[b51] Stevenson B. J. L. & Ollis J. W. D. L. Directed evolution of yeast pyruvate decarboxylase 1 for attenuated regulation and increased stability. Biochemistry. 47, 3013–3025 (2008). 18232643 10.1021/bi701858u

[b52] Andrews J. M. Determination of minimum inhibitory concentrations. J Antimicrob Chemother 48, 5–16 (2001). 11420333 10.1093/jac/48.suppl_1.5

[b53] Segel I. H. Enzyme kinetics: Behaviour and analysis of rapid equilibrium and steady state enzyme systems. Wiley and sons, USA (1993).

